# Electrical Remodeling and Low Voltage Areas in Atrial Fibrillation Patients with Functional Mitral Regurgitation: A Multicenter Evaluation

**DOI:** 10.31083/RCM26288

**Published:** 2025-03-18

**Authors:** Li-Jun Zeng, Xiao-Bo Pu, Xin Wei, Xi Wang, Ming-Yang Gao, Xue-Rong Sun, Cai-Hua Sang, Xing-Peng Liu, Mao Chen

**Affiliations:** ^1^Laboratory of Cardiac Structure and Function, Institute of Cardiovascular Diseases, West China Hospital, Sichuan University, 610041 Chengdu, Sichuan, China; ^2^Department of Cardiology, West China Hospital, Sichuan University, 610041 Chengdu, Sichuan, China; ^3^Department of Cardiology, Beijing Anzhen Hospital, Capital Medical University, 100069 Beijing, China; ^4^Heart Center, Beijing Chaoyang Hospital, Capital Medical University, 100069 Beijing, China

**Keywords:** atrial functional mitral regurgitation, atrial fibrillation, atrial fibrosis, voltage mapping

## Abstract

**Background:**

Atrial fibrosis may act as a substrate for atrial fibrillation (AF) and atrial functional mitral regurgitation (MR); thus, recognition is required to select the optimal therapeutic intervention.

**Methods:**

We examined clinical data from 1045 consecutive patients in three centers who underwent catheter ablation for persistent AF between 2020 and 2022. 75 patients met the moderate and severe MR criteria and completed a 1-year follow-up. Voltage mapping during the ablation procedure was reviewed to classify the extent of atrial fibrosis.

**Results:**

Significant atrial fibrosis was found in 34 patients (45.3%), and these patients had a higher prevalence of congestive heart failure (New York Heart Association (NYHA) II–III: 76.5% vs. 36.6%, *p* < 0.001) and an increased incidence of biatrial enlargement at baseline than the mild fibrosis group. At the 1-year post-ablation period, the entire cohort exhibited a decrease in left atrial size (41.6 ± 6.5 mm vs. 45.5 ± 5.3 mm, *p* < 0.001), and a significant reduction in MR was achieved in 70.7% of patients. The significant fibrosis group had a higher recurrence rate of atrial arrhythmias (55.9% vs. 22.0%, log-rank *p* = 0.002) and no significant change in atria size compared with baseline diameters (left atrium, 44.4 ± 6.4 mm vs. 47.2 ± 5.6 mm, *p* = 0.068; right atrium, 44.7 ± 11.2 mm vs. 46.7 ± 6.2 mm, *p* = 0.427).

**Conclusions:**

This study revealed a considerable proportion of significant fibrosis in patients with atrial functional MR and AF, leading to limited effectiveness in reducing atrial size following catheter ablation. Optimal intervention to reduce atrial size and recurrent arrhythmias in this population requires further investigation.

## 1. Introduction

Atrial functional mitral regurgitation (AFMR) is a newly recognized condition that occurs as a consequence of left atrial dilatation without organic mitral valve disease, typically in the setting of persistent atrial fibrillation (AF) [1]. This abnormality of atriogenic leaflet tethering differs from the mechanisms associated with left ventricular (LV) dilatation and dysfunction and has received much attention as an important cause of heart failure (HF) [2]. Gertz *et al*. [3] suggested that restoring sinus rhythm has a therapeutic effect on AFMR by reducing the enlarged left atrium (LA) and mitral annular dimension. However, 24% of patients who maintained sinus rhythm and over 80% of patients with recurrent AF still had significant mitral regurgitation (MR) at follow-up, suggesting that left atrial remodeling and dysfunction, which are indicators of underlying atrial myopathy, might contribute to AFMR in this population [4].

Previous studies have delineated the progression of atrial myopathy, characterized by atrial fibrosis and dysfunction, which contributes to the development of AF and subsequent stroke [5, 6]. Recently, researchers have identified the role of left atrial dynamics in MR and atrial dysfunction measured by echocardiography [4, 5]. These findings suggest the hypothesis that atrial fibrosis is responsible for persistent AF and MR.

Presently, no study has evaluated the extent of atrial fibrosis underlying AFMR and AF. Among the techniques for assessing atrial myopathy, electroanatomic mapping has become the standard for invasive substrate characterization through the geographic display of unipolar and bipolar signal amplitude data [7]. Thus, this study sought to retrospectively investigate the clinical outcomes of catheter ablation for patients with AF and AFMR and to evaluate the extent of atrial fibrosis using three-dimensional (3D) voltage mapping to discuss the selection of more favorable interventions in this patient population.

## 2. Methods

### 2.1 Study Population

We retrospectively reviewed all patients referred to 3 centers (West China Hospital, Beijing Anzhen Hospital, and Beijing Chaoyang Hospital) for catheter ablation of persistent AF between 2020 and 2022. Reports from transthoracic echocardiography performed within one week of cardiac catheterization were screened with the following inclusion criteria: (1) moderate or severe secondary MR; (2) LV ejection fraction (LVEF) >50%; (3) LV end-diastolic diameter <55 mm; (4) systolic LA anteroposterior diameter >35 mm; (5) normal leaflet anatomy and motion. Patients with intrinsic mitral abnormalities, including rheumatic, degenerative, congenital mitral valve disease, or extensive calcification involving mitral leaflets and annulus, were excluded from this study [8]. Other exclusion criteria were LV regional wall motion abnormality (assessed based on wall thickening and endocardial motion of myocardial segment in echocardiography) [9], myocardial infarction, or cardiac surgery within 3 months. Demographic and clinical information were prospectively obtained in all patients. All participants provided informed consent before the ablation procedure alongside authorization to use their data for research purposes. The study was conducted in accordance with the Declaration of Helsinki and approved by the Institutional Ethics Review Committees at West China Hospital, Beijing Anzhen Hospital, and Beijing Chaoyang Hospital.

### 2.2 Assessment of Cardiac Structure and Function

Transthoracic echocardiography was performed according to published guidelines [8, 9, 10]. LA anteroposterior systolic diameter was measured in the parasternal long-axis view, and right atrial (RA) transverse systolic diameter was measured in the apical four-chamber view. LVEF was calculated using the modified Simpson’s method. The severity of MR and tricuspid regurgitation (TR) was defined using a multiparametric approach, including assessing vena contracta width, effective regurgitant orifice area, and regurgitation volume. MR and TR were graded as none, mild, moderate, or severe.

### 2.3 Catheter Ablation and Voltage Mapping

Patients were on oral anticoagulation for at least 1 month before the procedure, and transesophageal echocardiography was performed to exclude LA thrombi. Percutaneous catheter ablation was performed without discontinuing anticoagulation. Electroanatomical mapping was performed using the CARTO 3 system and a 20-pole PentaRay catheter (Biosense Webster, Irvine, CA, USA). Voltage mapping was performed during sinus rhythm using a PentaRay catheter before ablation. Fifteen patients were in sinus rhythm before the procedure, and the remaining patients needed external electrical cardioversion to restore sinus rhythm. Circumferential pulmonary vein isolation (PVI) was applied using the technique and method described in previous study [11]. The cavotricuspid isthmus line was ablated if a typical atrial flutter was identified. Electrical cardioversion was performed for patients in which AF remained. Box lesion, also known as posterior wall isolation, was performed at the discretion of the physicians. The bilateral posterior PVI lines, roof, and floor ablation lines encircle the box lesion. Bidirectional conduction block was confirmed by performing differential pacing maneuvers.

Based on previous publications, we defined the presence of fibrosis as a region with a bipolar amplitude <0.5 mV [12, 13]. The extent of atrial fibrosis in this study was classified into normal (no detectable voltage area <0.5 mV), mild fibrosis (proportion of LA surface with severe fibrosis (<0.5 mV) of less than 50%); moderate fibrosis (proportion of LA surface with severe fibrosis (<0.5 mV) of more than 50%); severe fibrosis (diffuse diseased regions, **Supplementary Fig. 1**).

### 2.4 Follow-Up and Endpoints

The clinical follow-up consisted of physical examinations, transthoracic echocardiography, electrocardiogram, and 24-hour Holter monitoring performed 3 and 6 months after the procedure and every 6 months thereafter. Any cardiac symptoms that presented after ablation were considered an indication for 24-hour Holter monitoring. Recurrence of atrial arrhythmias was defined as any documented electrocardiographic episode of atrial arrhythmia lasting 30 seconds or longer with or without symptoms.

This study had two primary endpoints. The first was recurrent atrial arrhythmias associated with atrial fibrosis. The second primary endpoint was the change in the severity of MR associated with atrial fibrosis.

### 2.5 Data Analyses

Continuous variables are presented as the mean ± standard deviation and were compared using a two-tailed *t*-test. Categorical variables are presented as frequencies and percentages and were compared using a chi-square or Fisher’s exact test. Continuous variables were compared using a paired *t*-test for paired samples within groups. A *p*-value <0.05 was considered to be statistically significant. Statistical analyses were conducted using SPSS software version 26.0 (IBM Corporation, Armonk, NY, USA).

## 3. Results

### 3.1 Study Population

**Fig. 1.**
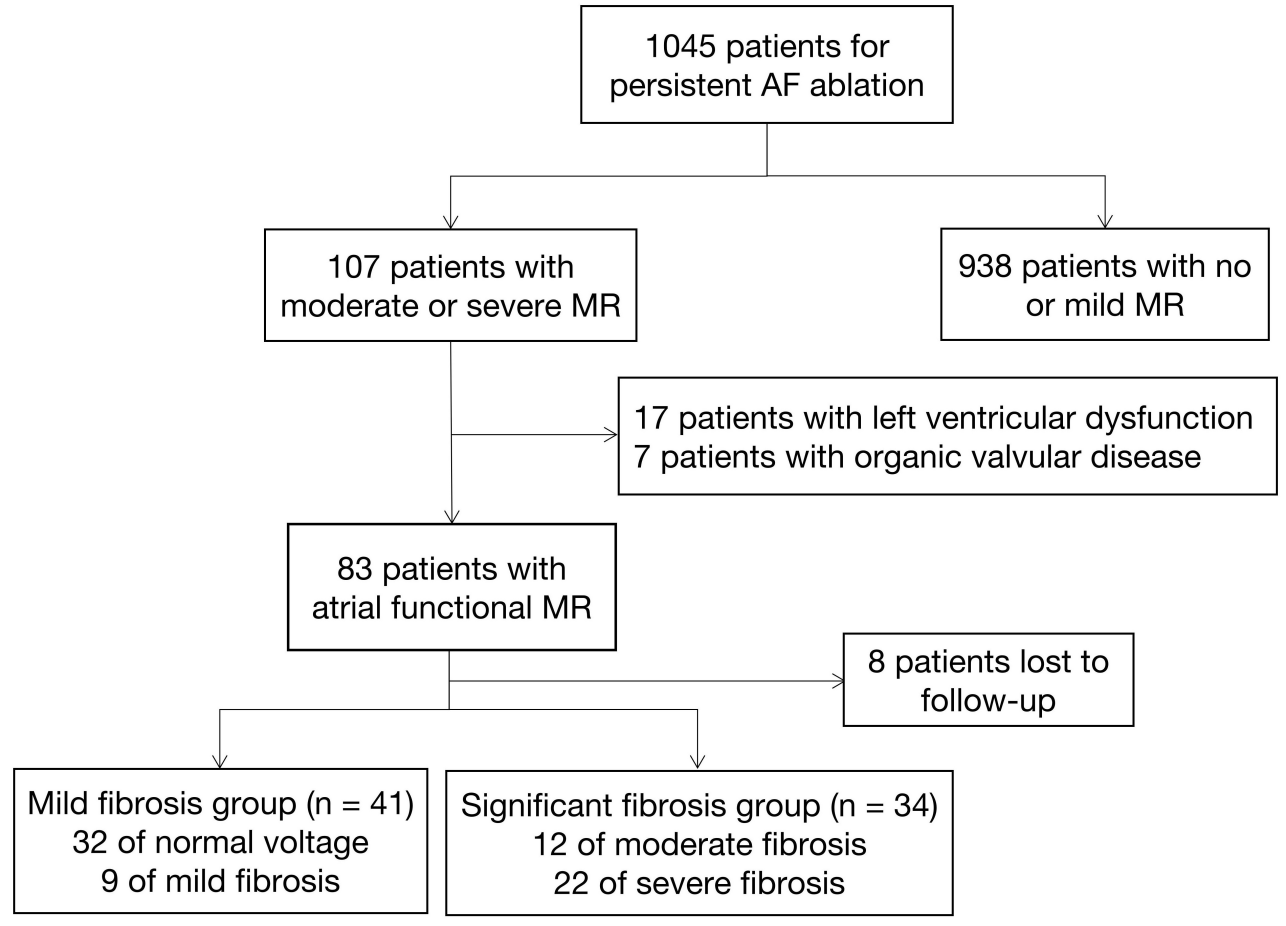
**Flowchart illustrating the mitral regurgitation cohort selections**. AF, atrial fibrillation; MR, mitral regurgitation.

The enrollment procedure for patients is illustrated in Fig. 1. In total, 1045 patients underwent catheter ablation for persistent AF at three centers between 2020 and 2022. After review, 83 patients met our criteria for moderate or severe MR; 75 patients remained for the 1-year clinical follow-up and were included in this study.

### 3.2 Voltage Mapping

Following endocardial 3D voltage mapping, nine patients with mild atrial fibrosis and 32 patients with normal atrial voltage were defined as the mild fibrosis group. Additionally, 12 patients with moderate and 22 with severe atrial fibrosis were defined as the significant fibrosis group. Beyond PVI, 14 patients (18.7%) underwent box lesions.

### 3.3 Baseline Clinical and Echocardiographic Characteristics

The baseline clinical and echocardiographic characteristics are presented in Table 1. In contrast to those with mild fibrosis, the group with significant fibrosis had a higher prevalence of congestive HF (New York Heart Association (NYHA) II–III: 76.5% vs. 36.6%, *p* < 0.001), more enlarged atria (LA: 47.2 ± 5.6 mm vs. 44.1 ± 4.7 mm, *p* = 0.012; RA: 46.7 ± 6.2 mm vs. 41.4 ± 5.8 mm, *p* < 0.001), and a higher tricuspid regurgitation peak gradient (TRPG, 34.0 ± 8.2 mmHg vs. 27.2 ± 7.9 mmHg, *p* = 0.001). Pacemakers were implanted in two patients due to sick sinus syndrome and in one patient owing to an atrioventricular conduction block.

**Table 1.** **Baseline and follow-up data**.

		Total (n = 75)	Mild fibrosis (n = 41)	Significant fibrosis (n = 34)	*p*-value
Gender (female)		39 (52.0%)	19 (46.3%)	20 (58.8%)	0.281
Age (year)		66.4 ± 10.8	64.2 ± 10.6	69.0 ± 10.6	0.052
BMI (kg/m^2^)		24.5 ± 3.1	25.0 ± 3.4	23.8 ± 2.6	0.104
AF duration (month), median (Q25, Q75)		12 (4, 36)	10 (4, 24)	18 (4, 36)	0.319
NYHA class					0.001
	I	34 (45.3%)	26 (63.4%)	8 (23.5%)	
	II	25 (33.3%)	7 (17.1%)	18 (52.9%)	
	III	16 (21.3%)	8 (19.5%)	8 (23.5%)	
Comorbidities					
	Hypertension	36 (48.0%)	18 (43.9%)	18 (52.9%)	0.435
	Diabetes mellitus	11 (14.7%)	6 (14.6%)	5 (14.7%)	0.993
	Prior stroke	7 (9.3%)	3 (7.3%)	4 (11.8%)	0.695
	CHA_2_DS_2_–VASc score	2.72 ± 1.58	2.15 ± 1.56	3.41 ± 1.33	<0.001
Echocardiography at baseline					
	Left atrium (mm)	45.5 ± 5.3	44.1 ± 4.7	47.2 ± 5.6	0.012
	Right atrium (mm)	43.8 ± 6.5	41.4 ± 5.8	46.7 ± 6.2	0.000
	LVEDD (mm)	48.9 ± 5.7	49.0 ± 5.5	48.7 ± 5.9	0.870
	LVEF (%)	57.2 ± 10.7	56.4 ± 11.3	58.1 ± 10.0	0.491
	Tricuspid regurgitation peak gradient (mmHg)	30.3 ± 8.7	27.2 ± 7.9	34.0 ± 8.2	0.001
Box lesion beyond PVI		14 (18.7%)	6 (14.6%)	8 (23.5%)	0.325
Atrial arrhythmia recurrence		28 (37.3%)	9 (22.0%)	19 (55.9%)	0.002
Echocardiography at 1 year					
	Left atrium (mm)	41.6 ± 6.5	39.2 ± 5.6	44.4 ± 6.4	0.012
	Right atrium (mm)	39.9 ± 9.6	36.7 ± 6.5	44.7 ± 11.2	<0.001
	LVEDD (mm)	47.5 ± 5.3	47.2 ± 6.0	47.9 ± 4.3	0.623
	LVEF (%)	61.6 ± 9.5	61.2 ± 9.9	62.2 ± 9.0	0.676
	Tricuspid regurgitation peak gradient (mmHg)	31.1 ± 13.6	28.0 ± 14.3	33.5 ± 13.0	0.263

AF, atrial fibrillation; BMI, body mass index; LVEDD, left ventricular end-diastolic diameter; LVEF, left ventricular ejection fraction; NYHA, New York Heart Association; PVI, pulmonary vein isolation.

### 3.4 Arrhythmia Recurrence

**Fig. 2.**
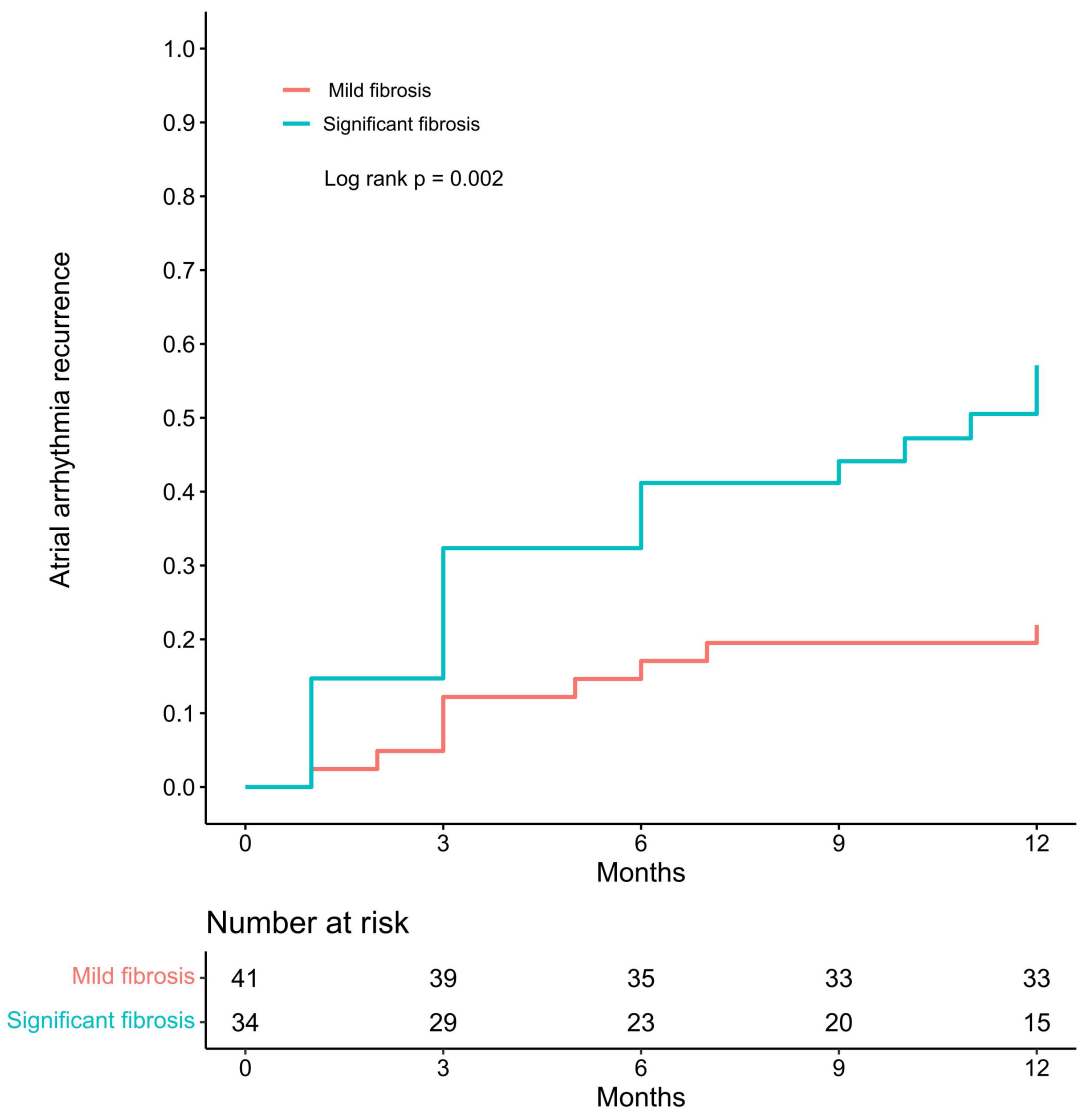
**Cumulative incidence curves of recurrence of atrial arrhythmia**.

Within the 1-year follow-up, 28 (37.3%) patients in the entire cohort had atrial arrhythmia recurrences, with 10 having atrial tachycardia (AT). The group with significant fibrosis had a higher incidence of recurrent atrial arrhythmias (55.9% vs. 22.0%, log-rank *p* = 0.002) than the mild fibrosis group (Fig. 2). In the mild fibrosis group, AF recurred in five patients, three of whom had paroxysmal episodes; three patients had recurrent AT. In the significant fibrosis group, AF recurred in 13 patients, four of whom had paroxysmal AF; the other six had recurrent AT.

### 3.5 Clinical Outcomes and Valvular Regurgitation

**Fig. 3.**
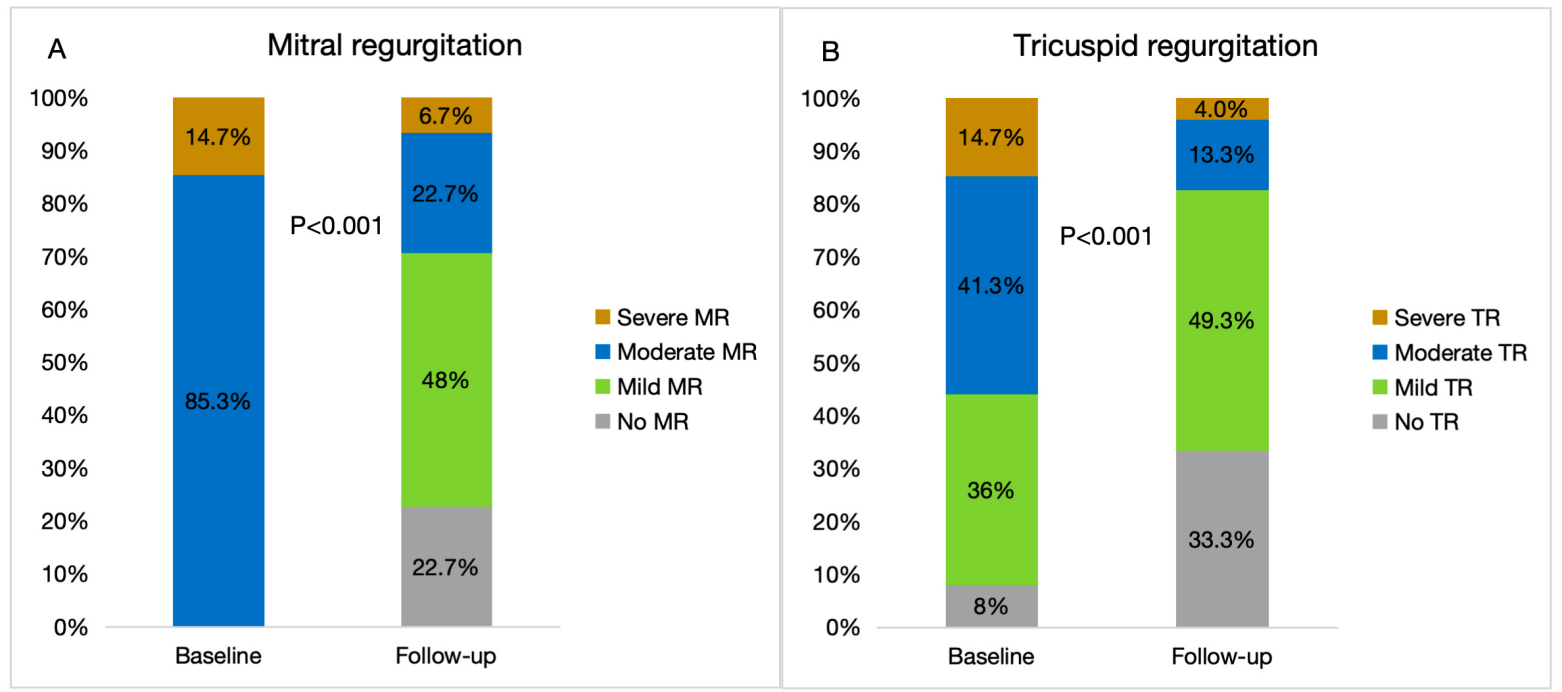
**Echocardiographic outcomes in the total cohort**. MR (A) and TR (B) severity at baseline and follow-up. MR, mitral regurgitation; TR, tricuspid regurgitation.

The atria size decreased in the entire cohort (LA: 41.6 ± 6.5 mm vs. 45.5 ± 5.3 mm, *p* < 0.001; RA: 39.9 ± 9.6 mm vs. 43.8 ± 6.5 mm, *p* = 0.008), as well as the severity of MR and TR (Fig. 3).

**Fig. 4.**
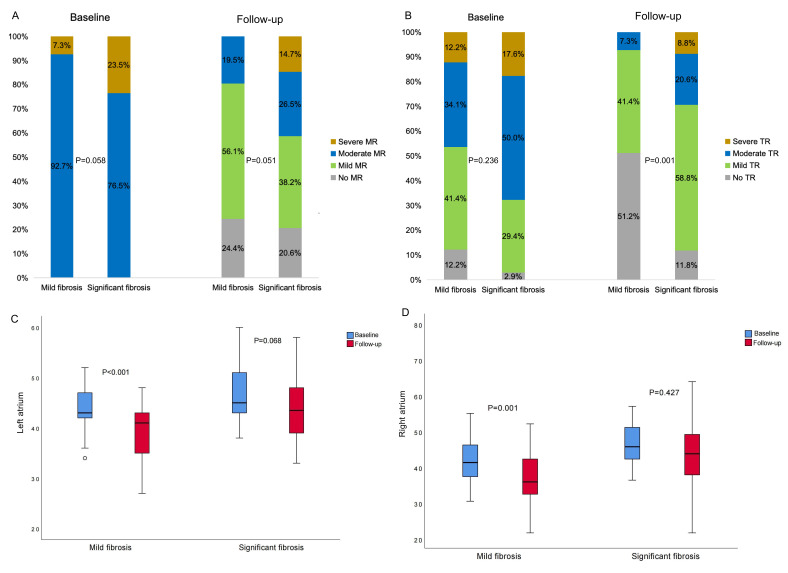
**Echocardiographic outcomes in mild and significant fibrosis groups**. MR (A) and TR (B) severity at baseline and follow-up. Left atrial (C) and right atrial (D) size at baseline and follow-up between groups. MR, mitral regurgitation; TR, tricuspid regurgitation.

At the follow-up visit, the group with significant fibrosis had a more enlarged LA size (44.4 ± 6.4 mm vs. 39.2 ± 5.6 mm, *p* = 0.012) and RA (44.7 ± 11.2 mm vs. 36.7 ± 6.5 mm, *p* < 0.001), and more severe TR (Fig. 4B) than the mild fibrosis group. In the significant fibrosis group, the atria size did not decrease after ablation (Fig. 4C,D), and 14 patients (41.2%) had moderate or severe MR at follow-up (Fig. 4A). LA size was not significantly different between the group with recurrent arrhythmias and the sinus rhythm group (42.9 ± 7.3 mm vs. 40.8 ± 5.8 mm, *p* = 0.185). In the multivariable regression analysis, residual moderate and severe MR was associated with female gender [odd ratio (OR) 4.336, 95% CI 1.340 to 14.029] and LA diameter (OR 1.113, 95% CI 1.016 to 1.220).

Of the 34 patients with significant fibrosis, one patient with recurrent AF underwent a stroke despite anticoagulant therapy, one patient with recurrent AT had a transient ischemic attack (TIA), and one patient died due to cerebral hemorrhage. Four patients were re-hospitalized for congestive HF; two of them were in sinus rhythm. One patient with mild fibrosis died due to non-cardiovascular disease.

## 4. Discussion

To our knowledge, this study is the first to provide evidence of a considerable proportion of significant atrial fibrosis underlying AFMR and AF, which could lead to reduced success following AF ablation and the need for further intervention for MR. In this multicenter study, moderate or severe AFMR was found in 7.9% of patients with persistent AF undergoing catheter ablation; meanwhile, 45.3% of this AFMR cohort had significant atrial fibrosis. Compared to the mild fibrosis group, the group with significant fibrosis had a higher prevalence of congestive HF at baseline, a higher incidence of recurrent atrial arrhythmias, and unreduced atria size after catheter ablation. In this group, rehospitalization for HF and stroke/TIA occurred in seven patients during the 1-year follow-up.

### 4.1 Prevalence and Prognosis of Atrial Functional MR

The incidence of significant AFMR was reported in 8.1% of patients with persistent AF, whereas the prevalence for patients with longstanding persistent AF (duration >10 years) was 28% [14]. The event-free rate for cardiac death or HF hospitalization was 53% at 24 months, and those with concomitant secondary TR had a worse prognosis, with an event-free rate of 27%. In our group with significant fibrosis, moderate and severe TR was found in 67.6% of patients at baseline and 29.4% at follow-up. The combination of AFMR and TR, which had the poorest prognosis, should receive greater therapeutic attention.

In a study involving hospitalized HF patients with AF, the prevalence of AFMR was 15.9% (30/189), and the all-cause mortality and HF rehospitalization rates over a median period of 273 days were 13.3% and 36.7%, respectively [15]. AFMR patients had a higher risk of cardiac death and HF readmission than HF patients without MR, irrespective of LVEF. Based on these observations, AFMR requires increased attention because of the associated high cardiac event rate and poor prognosis.

### 4.2 Clinical Insights for Mechanisms

The mechanisms underlying AFMR were reported previously. AF induces electrical and structural remodeling of the LA, leading to dilatation, which in turn causes enlargement of the mitral annulus. Mitral annular dilatation, atriogenic leaflet tethering, and insufficient leaflet remodeling also contribute to the pathogenesis of AFMR [1, 16]. Theoretically, restoration of sinus rhythm could reverse atrial remodeling and decrease MR. However, the extent of left atrial fibrosis is associated with a lower success rate following ablation [17].

The potential importance of atrial myopathy in AFMR is exhibited not only in electrical remodeling and the maintenance of AF but also in poor atrial systolic function assessed by strain measurements [4]. In cases of HF with preserved LVEF, atrial dysfunction plays the dominant role in the pathophysiology of functional MR, which reversely increases atrial tension and progressively leads to atrial dysfunction; the bidirectional relationship creates a vicious cycle [4].

### 4.3 Clinical Implications for Intervention

The recent 2021 ESC/EACTS guidelines for valvular disease recommend valve surgery or transcatheter edge-to-edge repair (TEER) for patients with severe MR who are consistently symptomatic despite standard medical therapy for HF [18]. Nevertheless, the guidelines did not divide the origin of secondary MR into ventricular and atrial dysfunction. Additionally, the recommendations in the 2020 Japanese guidelines, particularly for AFMR, are similar to the 2021 ESC/EACTS guidelines mentioned above, but with the recommendation that catheter ablation is reasonable for symptomatic patients with persistent AF and severe MR if successful ablation and maintenance of sinus rhythm can be expected [19]. In a retrospective study involving a small number of patients with AF and AFMR, catheter ablation was associated with a lower risk of readmission from HF and stroke compared to those treated with medical therapy [20]. However, the patients with AF recurrence exhibited no reduction in LA size and severity of MR after ablation [3]. Since a considerable proportion of increased remodeled atrial substrate exists in patients with persistent AF, catheter ablation might not be sufficient to reduce atrial size and MR severity through rhythm control, regardless of the ablation strategy. The recently released DACAAF II study revealed that fibrosis-targeted ablation plus PVI, compared with PVI alone, resulted in no significant difference in atrial arrhythmia recurrence [21]. The arrhythmogenic propensity of fibrotic tissue could not be eliminated by ablation, as atrial fibrosis was detected to be progressive and contributed to the recurrence of arrhythmias [22]. Combined with our study, these studies suggest atrial fibrosis may serve as a prognostic surrogate for more advanced disease. Moreover, these studies provide a rationale for investigating the optimal intervention for both atrial reversal remodeling and hemodynamic improvement.

TEER has been proven efficient in several small retrospective studies [23, 24, 25] and a registry study [26], with an acute and long-term reduction in the MR and NYHA functional class. However, current studies cannot definitely determine whether valve intervention is superior to ablation due to the limited sample size and the single-arm or retrospective design. A retrospective study reported that surgical valve repair combined with surgical ablation resulted in less readmission for HF and AF than catheter ablation alone over an 8-year follow-up period [27]. Another retrospective study found that surgical valve repair with concomitant ablation could benefit patients regarding recurrent MR, compared to surgical repair alone [28]. In this setting, well-designed comparative studies involving valve intervention, rhythm control, or combined strategies are needed for treating patients with AFMR and AF.

### 4.4 Limitations

The retrospective, observational design of our study makes it difficult to establish causal relationships. One of the main limitations was that mitral annular parameters and morphology were not measured by echocardiography or computed tomography (CT) scan, meaning the differences between subgroups could not be examined. Moreover, atrial myopathy involves the LA and the RA, manifested by a dilated RA diameter, TR, atrial tachyarrhythmias, and sinus node dysfunction [29]. Although we demonstrated an enlarged RA and significant TR in the study cohort, there was a lack of RA mapping during the ablation procedure, and we were unable to evaluate the extent of fibrosis in both atria. Further large-scale, prospective cohort studies are required to confirm and extend our findings on the relationships between AF, atrial fibrosis, and MR.

## 5. Conclusions

Our study revealed that significant fibrosis was present in a considerable proportion of patients with AF and AFMR and may supply the substrate for the limited effectiveness of rhythm control and atria reduction through catheter ablation. Given the small sample size in our study, further prospective studies are required for the optimal intervention in this patient population.

## Availability of Data and Materials

The data of this study are available on request from the corresponding author.

## Supplementary Material

Supplementary material associated with this article can be found, in the online version, at https://doi.org/10.31083/RCM26288.
