# Supplementary material for: Electrical Remodeling and Low Voltage Areas in Atrial Fibrillation Patients with Functional Mitral Regurgitation: A Multicenter Evaluation
Source: Rev Cardiovasc Med. 2025 Mar 18;26(3):26288. doi: 10.31083/RCM26288 (PMC11951295; doi:10.31083/RCM26288)
Supplement: Supplementary file 1 [file 2153-8174-26-3-26288-s1.docx]

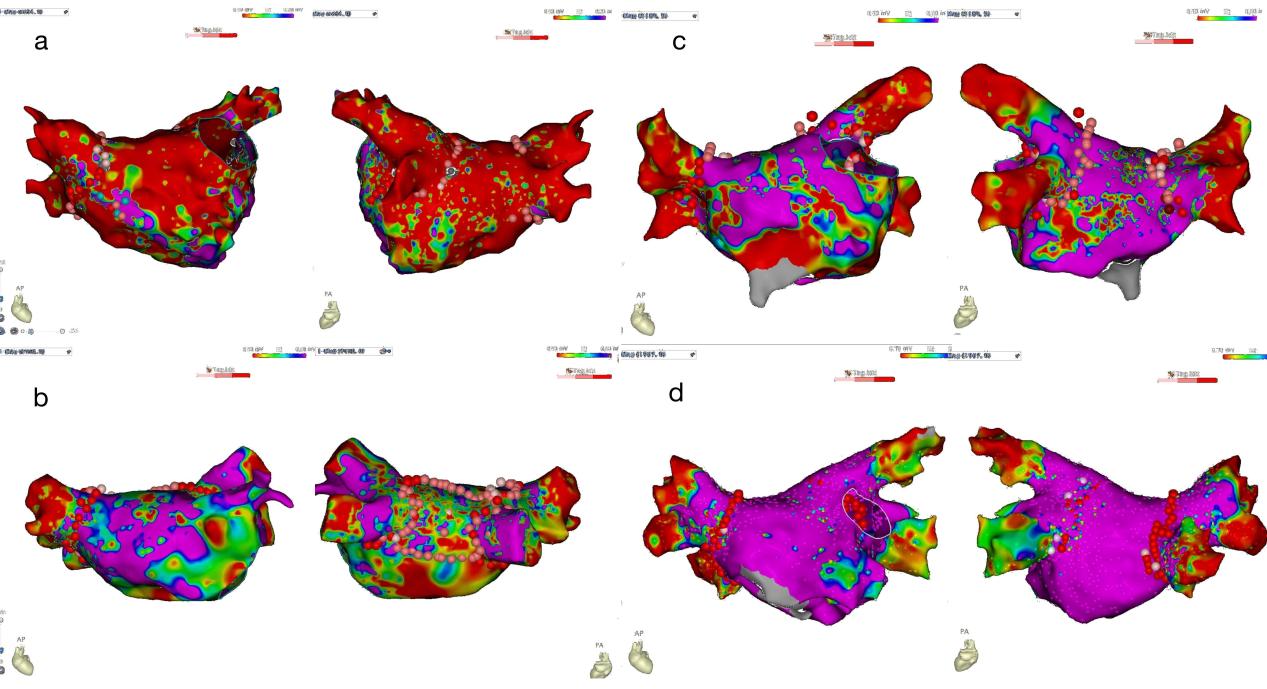


**Supplementary Fig. 1. Extent of atrial fibrosis by voltage mapping.** Voltage mapping of severe (a), moderate (b), mild (c), and normal (d) atrial fibrosis.
